# Retrospective Analysis of Patients With Prostate Cancer Initiating GnRH Agonists/Antagonists Therapy Using a German Claims Database: Epidemiological and Patient Outcomes

**DOI:** 10.3389/fonc.2018.00543

**Published:** 2018-11-27

**Authors:** Marie C. Hupe, Peter Hammerer, Miriam Ketz, Nils Kossack, Christiane Colling, Axel S. Merseburger

**Affiliations:** ^1^Department of Urology, University Hospital Schleswig-Holstein, Luebeck, Germany; ^2^Department of Urology, Academic Hospital Braunschweig, Brunswick, Germany; ^3^D-to-D Data to Decision AG, Hamburg, Germany; ^4^WIG2 GmbH—Scientific Institute for Health Economics and Health System Research, Leipzig, Germany; ^5^Ipsen Pharma GmbH, Ettlingen, Germany

**Keywords:** advanced prostate cancer, retrospective health service research, German claims database, GnRH agonist, GnRH antagonist, androgen deprivation therapy

## Abstract

**Objective:** The objective of this study was to obtain real-world information on gonadotropin-releasing hormone agonist/antagonist (GnRHa) therapy in patients with advanced prostate cancer (PCa).

**Materials and methods:** Anonymized, routine healthcare claims data from approx. 75 German statutory health insurance funds from 2010–2015 (*n* = 4,205,227) were analyzed. Patients had an enrolment of 1 year before GnRHa, 1 index quarter of initial GnRHa prescription and ≥2 years of follow-up.

**Results:** In total, 2,382 patients with PCa were eligible. The most frequent index therapy was leuprolide in 56.6%. The rank order of PCa comorbidity prevalence was consistent over time (% at index and 3-years of follow-up): hypertension (71.5; 85.0), hyperlipidemia (45.2; 60.8), cardiovascular disease *(*CVD) (35.7; 54.1), and diabetes (28.3; 36.2). Comparing pooled therapy classes (agonists, hybrids, and antagonist), no significant differences in the incidence of CVD or diabetes were observed. For hypertension, there was a significant increase for agonists (16.4%) compared to antagonists (6.9%, *p* = 0.022) and leuprolide hybrid group (11.6%, *p* = 0.006). During the follow-up period 23.9% of all PCa patients died. There were no significant differences concerning mortality rate and discontinuation rates between the cohorts. In total, 11.2% of all patients discontinued GnRHa after first prescription; the mean time to first switch to another GnRHa therapy was 100 days earlier for hybrids than for agonists (*p* = 0.016).

**Conclusion:** This comparative retrospective analysis provides real-world information about healthcare characteristics and treatment patterns, highlighting the impact of different GnRHa on clinical outcomes for patients with advanced PCa in Germany.

## Introduction

Prostate cancer (PCa) is the most common diagnosed cancer amongst men in Germany with 57,370 new diagnoses in 2014, and worldwide with more than one million new cases in 2012 (1, 2). Prostate cancer is a disease of the elderly: The risk for a 35-year-old man to develop PCa within the next 10 years is below 0.1%, whereas the risk of a 75-year-old man was found to be approximately 5% (1). Although PCa mortality has decreased in most countries — probably due to earlier diagnosis and improved therapy — PCa is still a leading cause of death and risk factors are poorly understood (2, 3). This emphasizes the need for further treatment optimization.

The dependence of PCa on hormones has been known for decades (4) and has led to testosterone suppression becoming the main treatment strategy in the palliative situation. Surgical castration remains the gold standard for androgen deprivation, but nonsurgical treatments allow intermittent therapy and have a lower psychological impact than orchiectomy (5). For men with locally advanced or metastatic prostate cancer (PCa), androgen deprivation therapy (ADT) is the mainstay treatment in order to reduce testosterone to levels obtained by surgical castration (<20–50 ng/dl, preferably <20 ng/dl) (6, 7). Different types of ADT exist, including GnRH agonists, GnRH antagonists and estrogens. GnRH agonists bind to GnRH receptors and mediate the same effect as GnRH: Increased release of LH and FSH leads to an increase in testosterone, which is called a flare-up. The continuous administration leads to continuous stimulation and thus to a downregulation of the receptor, so that LH, FSH and testosterone levels drop to the castration level (8). In contrast, GnRH antagonists (e.g., degarelix) block the GnRH receptor competitively and thereby prevent the release of LH and FSH. Thus, they inhibit signal transduction without causing a flare-up. Testosterone and PSA decline is achieved faster with GnRH antagonists than with agonists (9). Estrogens induce suppression of gonadotropin secretion in the pituitary gland and inhibit the production of androgens in the testicles. Due to their unfavorable safety profile with excessive cardiovascular and thromboembolic toxicity, estrogens are rarely used (10). Next-generation hormone manipulating substances are enzalutamide and abiraterone that are commonly used in the castration resistant setting (11). However, abiraterone has recently shown promising results in combination with an ADT in patients with metastatic hormone-sensitive PCa which has led to approval in this indication (12). Apalutamide is another new nonsteroidal antiandrogen that has been shown to improve metastasis-free survival in men with non-metastatic castration-resistant PCa and has been registered by the FDA (13).

Although ADT is the mainstay treatment in advanced and metastatic PCa, the existing guidelines contain only little information on the use of GnRH agonists and GnRH antagonists. Thus, the primary objective of this study was to analyze real-world information on healthcare characteristics and treatment patterns in patients with locally advanced or metastatic PCa dependent on the prescribed GnRH agonist/antagonist agents (GnRHa) in the first 3 years after initiation. The secondary objective was to compare the different agents regarding clinical implications and epidemiologic outcomes. GnRHa (substance classes) of interest were buserelin, goserelin, leuprolide (without hybrids), triptorelin (agonists), leuprolide hybrids (hybrids^1^), and degarelix (antagonist).

## Materials and methods

This study was a retrospective analysis using routine data from German statutory health insurances (GKV). As this study was an anonymized evaluation of health claims data, the study was exempt from ethical approval.

### Data source

Data were obtained from about 75 German statutory health insurances (GKV), mainly company health insurance funds (BKK) and guild health insurance funds (IKK) (14). The database contains anonymized data from more than six million insured persons. For analytical purposes, an anonymized and pseudonymized sample of this database was selected, which comprises all GKV insured patients, who were insured at least 1 day between 2010 and 2015. This sample is GKV-representative concerning age, sex, regions, and the morbidity in Germany.

### Study population

**Figure 1:**
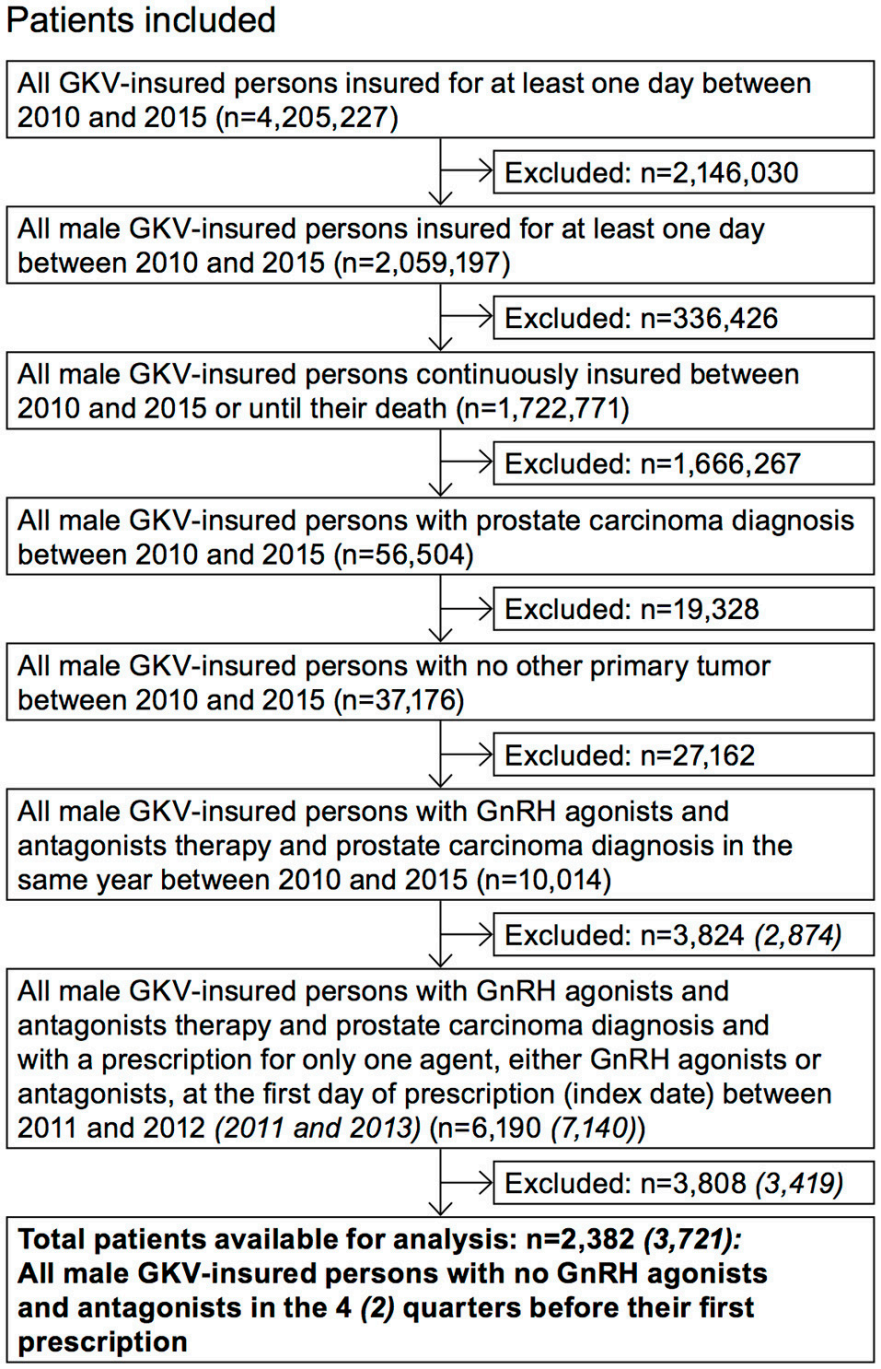
Selection of study population. In brackets: adjusted population.

Inclusion and exclusion criteria were applied in a stepwise manner to identify the desired study sample (Figure 1). Some selected questions were appropriately examined with a broader population (“adjusted population”). The selection criteria deviating from the study population are also shown in Figure 1.

### Study time periods

Data from patients insured at least 1 day between January 2010 through December 31, 2015 were used. The acquisition time interval for the first observed GnRHa treatment included January 1, 2011 through December 31, 2012 in order to get a pre-index period of 1 year and a follow-up period of 3 years. Each patient included into the study population has an individual study period: The timeframe of each individual study period is determined by the individual GnRHa prescription date (index date), which is identified within the acquisition period from 2011 through 2012. Additionally, a timescale before (pre-index period) and after (follow-up period) the index date is necessary to evaluate the first GnRHa treatment and to analyze the treatment pathway in the first 3 years after (including) initiation of different GnRHa uses. The pre-index period lasts from January 1, 2010 through December 31, 2011 and the follow-up period starts January 1, 2013 and ends December 31, 2015.

The higher number of patients in the adjusted population results from a longer index period (2011 to 2013) and a shorter pre-index period (2 quarters) compared to the study population (Figure 1). The follow-up period of the adjusted population was shorter with only 2 years.

### Cohort selection

After applying the inclusion and exclusion criteria, the study population was divided into six patient cohorts based on their first prescription of the following GnRHa drugs: buserelin, goserelin, leuprolide (without leuprolide hybrids), triptorelin, leuprolide hybrids, and degarelix.

### Treatment patterns

The following treatment patterns were defined:

1. *Switch:* A therapy switch was defined as change to a different agent in the follow-up period.
2. *Continuation:* A therapy continuation was defined as a further prescription of an agent.
3. *Discontinuation:* A therapy discontinuation was defined as a period of >6 months from the date of the last prescription to the end of the follow-up period without any further prescription or if the patient died within the follow-up period. The date of therapy discontinuation was defined as day of death or 6 months after the last prescription date.
4. *Interruption:* A therapy interruption was defined as a period of >6 months between two prescriptions. The date of therapy interruption was defined as the first day 6 months after the last prescription. The duration of the therapy interruption was calculated from the first day of therapy discontinuation and the date of the next agent prescription.
5. *Treatment phase:* The beginning of a therapy or treatment phase was defined as the first prescription of an agent. The end of a therapy or treatment phase was marked as a switch, as a discontinuation or as an interruption.

### Statistics

Results were evaluated applying descriptive analyses. Chi-square testing was used for categorical variables. The Wilcoxon rank sum test was used for continuous. A *p*-value of < 0.05 was considered statistically significant.

## Results

### Patients

After applying the inclusion and exclusion criteria, a total of 2,382 patients were identified for the analysis with index GnRHa therapy in 2011/2012 (Figure 1) and were stratified into six main cohorts according to their treatment. Patient characteristics are summarized in Table 1. Mean age was approx. 75 years. The annual PCa prevalence was between 2.0 and 2.3% (2010–2014); the highest rates were found in the age group of 80–89-year-old patients (about 12%). At initial GnRHa treatment, 70% of patients showed no lymph node involvement or metastases. Patients initially receiving degarelix were the youngest (mean age 72 years) and comprised the highest proportion of those with metastases (38%; *p* = 0.002).

**Table 1:** Demographic data of the study population and patients per index therapy (cohorts) in index years 2011/2012.

		**Cohorts**					
**GnRHa index therapy**	**Total population (*N* = 2,382)**	**Buserelin (*N* = 244) 10.24%**	**Goserelin (*N* = 119) 5.00%**	**Leuprolide (*N* = 1,347) 56.55%**	**Triptorelin (*N* = 308) 12.93%**	**Leuprolide hybrids (*N* = 312) 13.10%**	**Degarelix (*N* = 52) 2.18%**
Age (years), median	75	76	75	75	75	76	74
**AGE GROUP (%)**							
0–39 40–49 50–59 60–69 70–79 80–89 90+	0.00 0.08 3.61 17.63 50.13 26.66 1.89	0.00 1.02 2.46 13.93 58.20 23.36 1.02	0.00 0.00 2.10 22.69 41.18 31.93 2.10	0.00 0.07 4.01 17.89 49.15 26.80 2.08	0.00 0.00 1.62 19.81 53.57 23.70 1.30	0.00 0.00 3.53 15.06 47.76 31.41 2.24	0.00 0.00 11.54 19.23 51.92 15.38 1.92
**STAGE OF PCA DISEASE (%)**							
Total	100	100	100	100	100	100	100
N0 and M0^*^	70.07	68.85	63.87	71.05	72.08	70.51	50.00
N1 and M0^*^	3.90	3.69	0.84	4.31	3.90	3.85	1.92
N0 and M1^*^	6.51	4.92	10.08	6.83	4.87	6.09	9.62
N1 and M1^*^	19.52	22.54	25.21	17.82	19.16	19.55	38.46

* *The findings are based on the coding according to ICD (International Statistical Classification of Diseases and Related Health Problems) and do not result from clinical histopathological TNM staging. No lymph node metastases (= N0); no distant metastases (= M0); lymph node metastases (= N1); distant metastases (= M1)*.

### Comedications and comorbidities

Most commonly used drugs taken parallel to the index GnRHa were agents manipulating the renin-angiotensin system (44.3%) and beta-adrenergic antagonists (28.6%). Comedication rates did not differ significantly between the cohorts.

**Figure 2:**
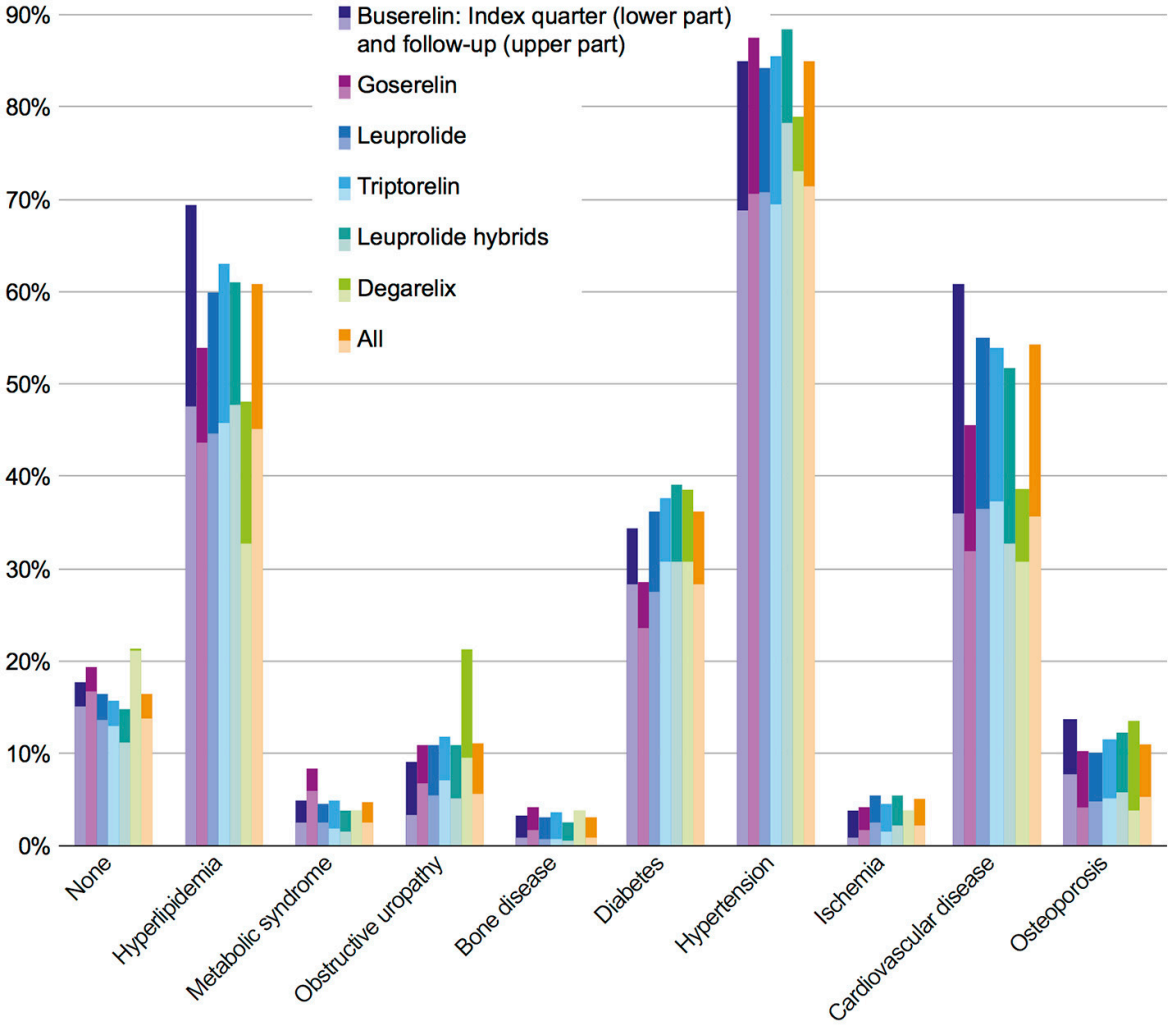
Prevalence of comorbidities in index quarter and follow-up period in all investigated cohorts. Each bar indicates prevalence of comorbidities in index period (lower part) + 3 year follow-up (upper part).

Most frequent comorbidities in the study population in index quarter at the time of GnRHa initiation were hypertension (71.5%), hyperlipidemia (45.2%), cardiovascular diseases (CVD) (35.7%), and diabetes (28.3%). The rank order of the comorbidity prevalence stayed the same within the first 3 years. In the follow-up period 85.0% of the patients suffered from hypertension, 60.8% from hyperlipidemia, 54.1% from CVD, and 36.2% from diabetes mellitus. Comorbidity prevalence rates in the index quarter and in the follow-up period are shown in Figure 2. The comorbidity results independent of the GnRHa treatment were comparable to 5-years-prevalence rates of comorbidities (2010–2014) of the benchmark population defined as “All male GKV-insured persons with prostate carcinoma diagnosis between 2010 and 2015 and no other primary tumor.”

**Figure 3:**
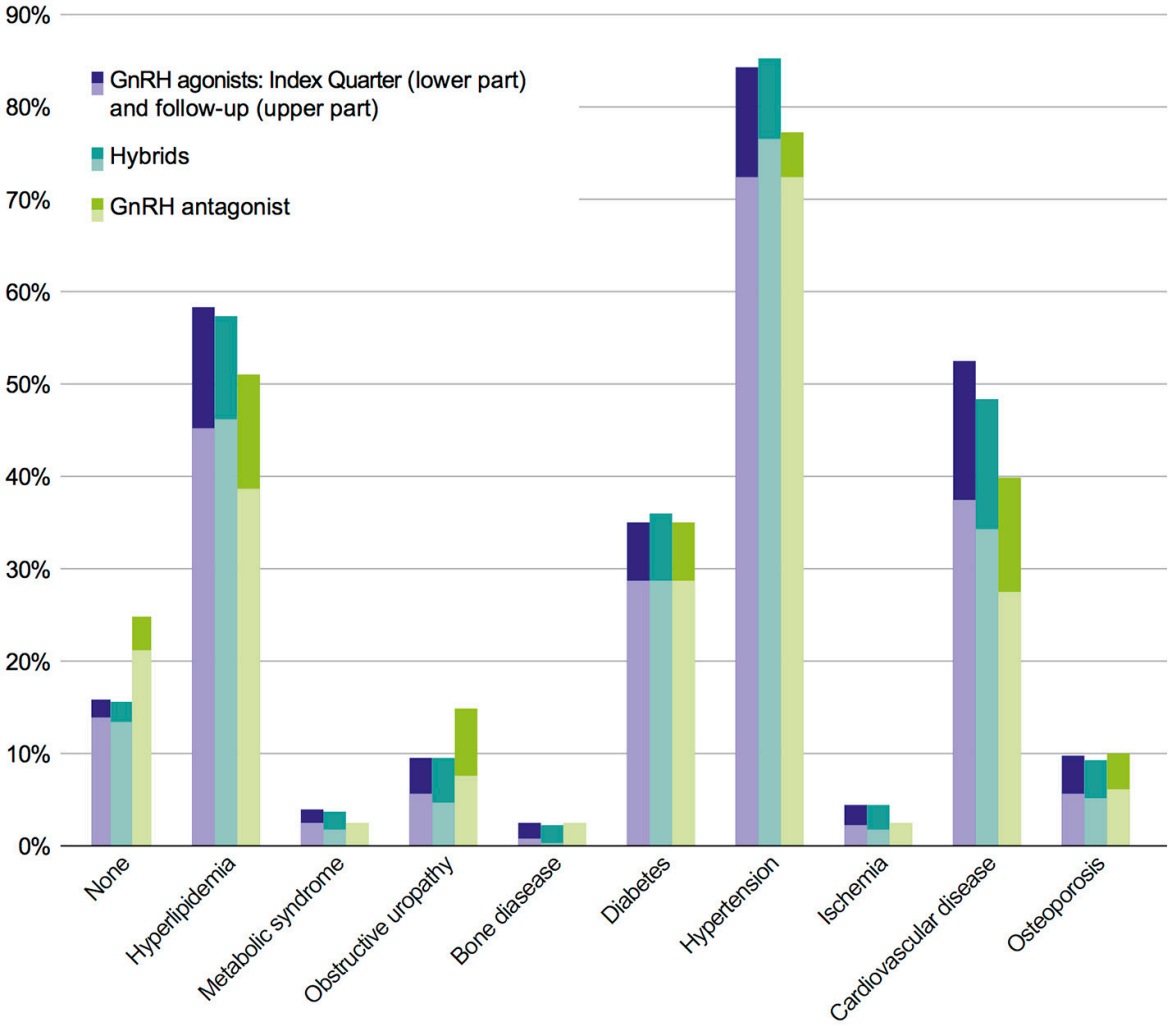
Prevalence of comorbidities of the adjusted population in pooled therapy classes. Each bar indicates prevalence of comorbidities in index period (lower part) + 2 years follow-up (upper part).

In the study population, no statistically significant differences in comorbidities were observed between the six cohorts of interest, neither at index prescription nor after a 3-year follow-up. In order to answer the question whether or not comorbidities are dependent on therapy classes, an adjusted population with a higher patient number was defined (Figure 1); this adjusted study population includes 3,721 patients. The comorbidity rates of the adjusted population were similar to the results of the study population with the most prevalent comorbidities being hypertension, hyperlipidemia, CVD, and diabetes (Figure 3).

Significant differences between GnRH agonists and the GnRH antagonist were observed regarding the relative increase of hypertension and obstructive uropathy (Table 2): From index to follow-up period, the growth rate of hypertension was significantly higher in the GnRH agonist class compared to the antagonist (*p* = 0.022). The relative increase of obstructive uropathy was significantly lower for GnRH agonists compared to the antagonist (*p* < 0.001). Similar to the comparison of GnRH agonists and the antagonist, relative increases of hypertension and obstructive uropathy were significantly different between GnRH agonists and hybrids (Table 2): Regarding hypertension, the relative growth rate from index to follow-up period was significantly higher for GnRH agonists than for hybrids (*p* = 0.006). The relative increase of obstructive uropathy was significantly lower for GnRH agonists compared to hybrids (*p* < 0.001).

**Table 2:** Relative growth rates from index to follow-up for hypertension, CVD, obstructive uropathy, and diabetes (adjusted study population: therapy classes, *N* = 3,721; *in brackets: only significant p-values are shown*).

	**Relative growth rate (%)**			
	**Hypertension**	**Obstructive uropathy**	**CVD**	**Diabetes**
GnRH agonists *N* = 3,149	16.40*(p = 0.022 vs. antagonistp = 0.006 vs. hybrids)*	70.11*(p < 0.001 vs. hybrids and antagonist)*	40.26	22.31
Hybrids *N* = 491	11.56	100^*^	40.72	25.00
GnRH antagonist *N* = 81	6.90	100^*^	45.45	21.74

* *For hybrids and GnRH antagonists, there was a doubling in obstructive uropathy during the follow-up period, i.e., a relative growth rate of 100%*.

Although significant differences were observed between therapy classes regarding hypertension, no significant differences have been observed in the relative increase of CVD and diabetes from index quarter to follow-up period between GnRH antagonist compared to GnRH agonists and hybrids. No significant difference in the absolute comorbidity rate of obstructive uropathy between GnRH agonists (9.4%), hybrids (9.4%), and antagonist (14.8%) was observed, although a (not statistically significant) trend was found in favor of agonists and hybrids.

### Mortality rate

Almost 1 quarter (23.9%) of all patients with PCa died during GnRHa therapy (*n* = 570) in the follow-up period. The triptorelin cohort had the lowest mortality rate with 22.1% (*n* = 68), while the goserelin cohort had the highest mortality rate with 29.4% (*n* = 35). However, there were no significant differences between the six cohorts of interest. Median and mean number of days until death as well as mortality rates are shown in Table 3.

**Table 3:** Mean and median days until death (follow-up period) and mortality rate (index quarter + follow-up period).

	**Days until death**			**Mortality rate *(all p = ns)***
**Therapy cohort**	***N***	**Mean**	**Median**	**%**
All	570	534.09	522.00	23.93
Buserelin (*N* = 244)	54	579.37	592.50	22.13
Goserelin (*N* = 119)	35	515.60	535.00	29.41
Leuprolide (*N* = 1,347)	315	527.93	517.00	23.39
Triptorelin (*N* = 308)	68	523.87	477.50	22.08
Leuprolide hybrids (*N* = 312)	83	535.07	535.00	26.60
Degarelix (*N* = 52)	15	584.60	534.00	28.85

### General GnRHa-prescription

On average, the first therapy phase lasted 476.4 days and 3.7 GnRHa prescriptions were given per patient. Nearly 90% of all PCa patients received one or more further GnRHa prescription(s) after the index therapy. In contrast, about 11.2% of the study population discontinued their GnRHa therapy after one initial drug prescription for at least 3 following years.

### Switches

**Figure 4:**
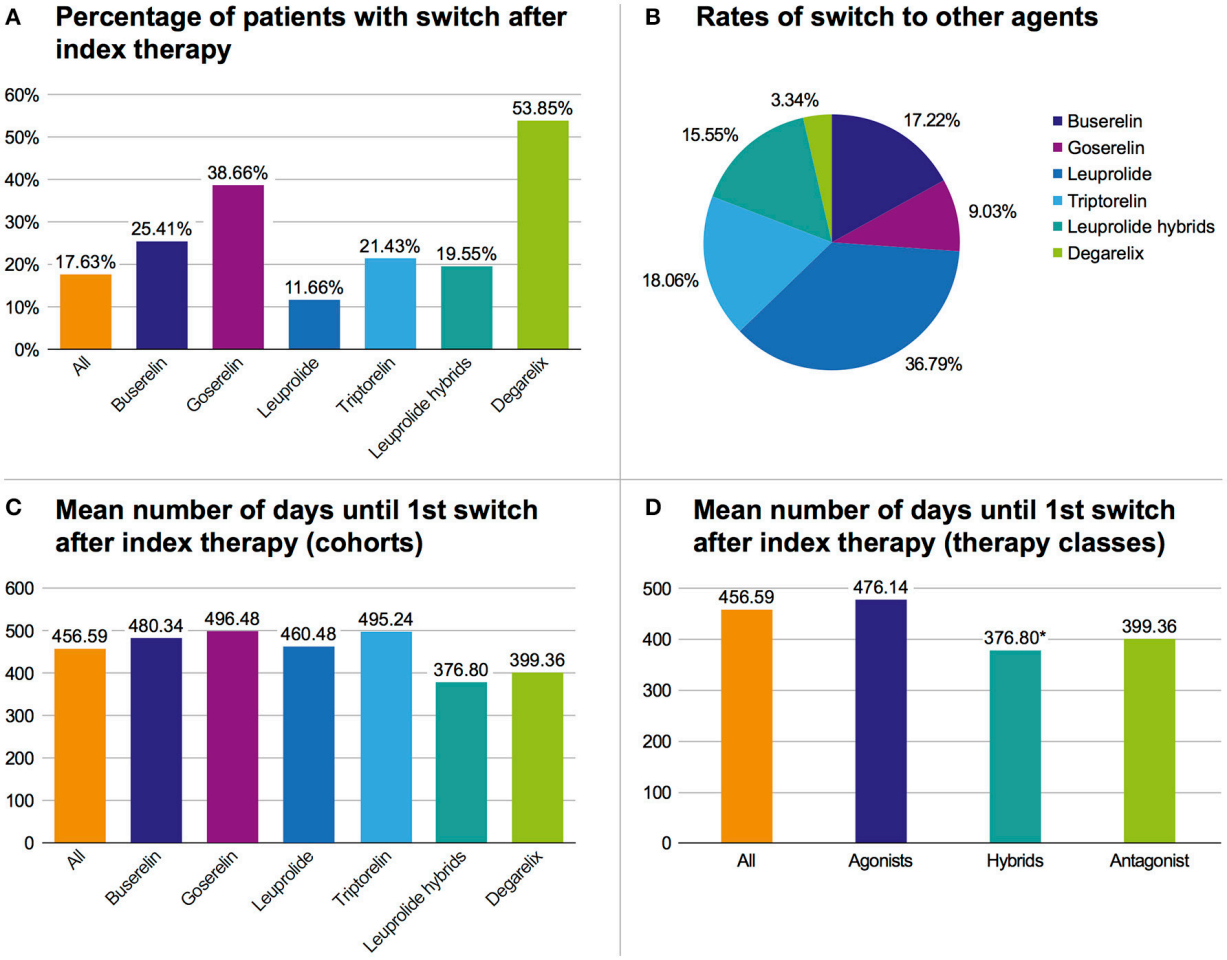
Overview of therapy switches. **(A)** Percentage of patients with switch after index therapy. **(B)** Rates of all switches to one of the six cohorts. **(C)** Mean number of days until first switch after index therapy based on cohorts. **(D)** Mean number of days until first switch after index therapy based on pooled therapy classes, *p < 0.05 vs. agonists.

Regarding all patients from the study population, 17.6% switched their initial GnRHa therapy within 3 years and 82.4% adhered to their initial index GnRHa class. There was a higher switch rate in the degarelix and the goserelin groups than in the triptorelin cohort. However, in the triptorelin cohort the switch rate was higher than in the leuprolide index population (Figure 4A). Regarding all switches of the study population in the follow-up period, most switches were to leuprolide (36.8%), followed by switches to triptorelin (18.1%) (Figure 4B). On average, the treatment time with the index GnRHa agent prior to a (first) switch was 456.6 days (median: 398.5 days [range: 308.0–474.5]). Patients treated with leuprolide hybrids switched faster (mean: 376.8 days) than patients treated with any other agent (mean of all: 456.6 days) (Figure 4C).

In pooled therapy classes, the first switch in the hybrids therapy class occurred significantly (*p* = 0.016) faster than in the GnRH agonist class (Figure 4D) (mean: 376.8 days vs. 476.1 days).

In all therapy populations (except leuprolide), the highest switch rate was to leuprolide (>50%). Within the leuprolide index population, patients mostly switched to triptorelin and hybrids (29.9% each).

### Discontinuation and interruptions

In total, 58.2% of the patients from the study population who started with a GnRHa therapy discontinued their therapy within 3 years. There were no significant differences concerning the discontinuation rates between the six index therapy cohorts. On average, therapy was discontinued 526.9 days after starting the first GnRHa therapy (including death). The most frequent therapy discontinuations (including death) occurred after leuprolide prescriptions (56.0%), followed by discontinuations after triptorelin (14.4%), leuprorelin hybrids (13.0%), and buserelin (10.8%).

Almost 40% of all patients had at least one therapy interruption. There were no significant differences in the interruption rates between triptorelin and the other cohorts with exception of leuprolide hybrids. The rate of patients with at least one GnRHa treatment interruption was significantly lower in the leuprolide hybrids cohort (27.9%) than in the triptorelin cohort (38.6%) (*p* = 0.004). With 63.8% most of the interruptions occurred after leuprolide, followed by interruptions after triptorelin (12.5%), leuprolide hybrids (9.1%), and buserelin (9.1%).

### Additional therapeutic options

Within three years after initial treatment with GnRHa agents, 14.3% of the study population were additionally treated with anti-androgens. Within the follow-up period, 6.5% of the patients were given abiraterone^2^, 5.2% were treated with a radiotherapy, 5.2% were given docetaxel^3^ chemotherapy, 2.9% underwent a radical prostatectomy, and 1.9% received enzalutamide^4^. Other additional therapeutic options including orchiectomy, chemotherapy with cabazitaxel^5^ and radionuclide therapy were negligible and pelvic lymphadenectomy was not performed in the cohort. The initiation of additional therapies in the study population during the follow-up period was analyzed to show the adherence rate to ADT, despite onset of new treatments. At start of a new drug therapy, the additional administration of GnRHa was discontinued in 8 to 27% of the patients (incl. death) (Table 4).

**Table 4:** Initial use of additional therapies during follow-up *(Italic and in brackets: % of study population)*.

**Therapy in addition to GnRHa treatment^a^ Total population (*N* = 2,382)**	**Proportion of patients with (≥ 1) continuing GnRH treatment (%)**	**Proportion of patients with switch of GnRH agent (%)**	**Proportion of patients with GnRH treatment interruption (%)**	**Proportion of patients with discontinuation (including death) (%)**
Anti-androgen therapy *N* = 340 *(14.27)*	67.65 *(9.66)*	8.53 *(1.22)*	11.47 *(1.64)*	**7.65** ***(1.09)***
Docetaxel *N* = 124 *(5.21)*	62.10 *(3.23)*	4.03 *(0.21)*	9.68 *(0.50)*	**20.16** ***(1.05)***
Cabazitaxel *N* = 20 *(0.84)*	60.00 *(0.50)*	0 *(0)*	0 *(0)*	**26.66** ***(0.17)***
Abiraterone *N* = 155 *(6.51)*	58.71 *(3.82)*	5.81 *(0.38)*	10.97 *(0.71)*	**18.06** ***(1.18)***
Enzalutamide *N* = 45 *(1.89)*	51.11 *(0.97)*	0 *(0)*	11.11 *(0.21)*	**24.44** ***(0.38)***
Radiotherapy *N* = 123 *(5.16)*	40.65 *(2.10)*	4.07 *(0.21)*	10.57 *(0.55)*	**43.9** ***(2.27)***
Prostatectomy *N* = 69 *(2.90)*	21.74 *(0.63)*	7.25 *(0.21)*	7.25 *(0.21)*	**59.42** ***(1.72)***

a *Surgical hormone deprivation (orchiectomy) was documented in 15 patients, pelvic lymphadenectomy in 0 patients, radionuclide therapy in 5 patients*.

## Discussion

This health service research aimed to obtain and analyze real world information on the therapy of patients with advanced PCa with GnRH agonists, hybrids, and antagonists between 2010 and 2015. Seventy percent of patients with prescribed GnRHa therapy showed no involvement of lymph node metastases or distant metastases. The rationale of GnRHa therapy in these patients may originate from the high-risk profile based on medical parameters not reflected in this database. In another real-world study in Germany (CAPRIS), approximately 60% of patients without lymph node involvement or metastases under ADT presented with high-risk PCa according to the d'Amico criteria (15).

In this current research, hypertension was observed to be the most frequent comorbidity with highest growth rates in the class of GnRH agonists. However, no statistically significant differences were found in the relative growth rates of CVD from index quarter to follow-up period between GnRH agonists compared to antagonists and hybrids. ADT increases cardiovascular morbidity in men with PCa (16). Since the potentially different effects of GnRH agonists and antagonists on CVD risk are still controversially discussed (17–20), one aspect of this analysis was the comorbidity rate, in particular CVD comorbidity, among GnRH agonists and the antagonist. However, due to the small number of patients in the degarelix cohort, the results do not allow any conclusion and more evidence is needed. A nationwide population-based cohort study based on French health insurance data from 2010 to 2013 investigated the effect of ADT on cardiovascular risk (21). From the patients included, 24,846 received a GnRH agonist and 1,273 were treated with an antagonist. They found no significant association between GnRH agonists and antagonists regarding myocardial infarction and ischemic stroke (adjusted HR 1.2, 95% CI [0.7–2.1]) Thus, Scailteux et al. concluded that the probability of a clinically meaningful difference comparing GnRH agonists and antagonists with regard to their effect on cardiovascular risk appears rather low (21).

Another comorbidity reducing survival of PCa patients is obstructive uropathy (22). The present study showed a significantly lower growth rate in obstructive uropathy when patients were treated with GnRH agonists compared to antagonists and hybrids.

Triptorelin had the lowest mortality rate of all cohorts of interest, although there were no statistically significant differences. This tendency is also reflected in a head-to-head trial with 284 patients showing, however in an exploratory analysis, a significantly higher 9-month survival rate for triptorelin than for leuprolide (97.0% vs. 90.5%; *p* = 0.033) (23). A positive trend for triptorelin was also mentioned in the work of Uttley et al. presenting a summary of the Evidence Review Group (ERG) report on the company's submission (CS) for degarelix (24). The ERG report is an important part within the NICE Single Technology Appraisal process, which critically evaluates the clinical and cost-effectiveness evidence of a substance based on the CS. A network meta-analysis presented in the CS has shown no statistically significant differences for overall survival between degarelix, leuprolide and goserelin, but a lower mortality for triptorelin compared to degarelix (24). Among most urologists, it is generally assumed that ADT is interchangeable, without any differentiation between individual GnRHa substances. Although their mode of action, i.e., medical castration, is comparable, the present study shows variabilities, thus supporting the following statement from Uttley et al. (24): “The ERG considered the assumption that the LHRH agonists goserelin, leuprorelin, and triptorelin are clinically equivalent to be unproven and therefore inappropriate.”

In this claims data analysis, patients treated with leuprolide hybrids switched significantly faster than patients treated with agonists. Due to missing clinical parameters in GKV data, it is unknown whether this is due to differences of substances handling or disease progression. An analysis from four studies with a leuprolide hybrid indicated that efficacy and safety of the hybrid is comparable to leuprolide (25). However, the authors stated that in two long-term studies the number of patients was low, limiting the significance of their results.

The proportion of switches was lowest (3.3%) to the antagonist (degarelix) with regard to all switches. Possible reasons include the low number of patients in the degarelix cohort and a lack of data regarding the efficacy of the treatment sequence GnRH agonist followed by antagonist.

Additional therapeutic options include the newer substances enzalutamide and abiraterone. In total, 18.1% of the patients receiving abiraterone and 24.4% of the patients receiving enzalutamide in addition to GnRHa treatment discontinued their ADT with GnRHa (incl. death). Pivotal trials of both enzalutamide and abiraterone conducted with maintaining ADT showed the effectiveness of continued ADT (26, 27).

The analysis was based on German statutory health insurance data (secondary data) whose original function is reimbursement of health care cost. This may have affected the generalizability of results. This study has the following limitations: (1) There was no claim of completeness regarding medical and pharmacy service. Every outcome that was irrelevant for reimbursement may not have been coded which can lead to the under-representation of certain health outcomes or can underestimate the prevalence of conditions. (2) The quality of claims data was dependent on the individual quality of coding for billing purposes and on the existing classification systems. (3) Use of claims data precludes factors like clinical information (e.g., BMI, smoker status, laboratory findings, cancer risk classes) and patient reported subjective outcomes. The analysis may therefore have failed to detect clinically important aspects and differences. (4) The number of patients in the analyzed GnRHa groups vary considerably.

## Conclusions

The results of this comparative retrospective analysis provide real-world information about the current use of different ADT options for patients with locally advanced or metastatic PCa in Germany. The findings indicate differences in treatment patterns as well as changes in clinical outcomes for the GnRH agents investigated. These epidemiological results may aid urologists in offering more suitable individual ADT options for patients with advanced PCa. However, this healthcare study does have various limitations which must be considered when interpreting the results.

## Ethics statement

As this study was an anonymized evaluation of health claims data, the study was exempt from ethical approval.

## Author contributions

CC, MK, PH, and AM: Study concept, Study design. MK, NK, and CC: Data acquisition, Statistical analysis. MH, AM, PH, MK, CC, and NK: Quality control of data and algorithms. MH, CC, AM, PH, and MK: Data analysisand interpretation, Manuscript preparation. CC, MH, and MK: Manuscript editing. MH, AM, and PH: Manuscript review.

### Conflict of interest statement

MH: travel grant and honoraria from Ipsen; PH: speaker/advisor for Janssen, Ipsen, Takeda, and Sanofi; CC: Employee of Ipsen Pharma GmbH, Ettlingen; AM: speaker/advisor for Astellas, BMS, Merck, Ferring, Roche, Janssen, Ipsen, Takeda, and Sanofi. The remaining authors declare that the research was conducted in the absence of any commercial or financial relationships that could be construed as a potential conflict of interest.

## Footnotes

^1^In Germany, two GnRH products have a so-called hybrid approval. A hybrid marketing authorisation is an authorisation which refers to an original product; however, the substance does not meet all the criteria for a generic medicinal product and therefore additional pre-clinical and clinical data must be submitted.

^2^ATC code: L02BX03, drug approval in the German Statutory Health Insurance (GKV) market in 2011

^3^ATC code: L01CD02, drug approval in the German Statutory Health Insurance (GKV) market in 2004

^4^ATC code: L02BB04, drug approval in the German Statutory Health Insurance (GKV) market in 2013

^5^ATC code: L01CD04, drug approval in the German Statutory Health Insurance (GKV) market in 2011
